# Accumulation of Tau Protein, Metabolism and Perfusion—Application and Efficacy of Positron Emission Tomography (PET) and Single Photon Emission Computed Tomography (SPECT) Imaging in the Examination of Progressive Supranuclear Palsy (PSP) and Corticobasal Syndrome (CBS)

**DOI:** 10.3389/fneur.2019.00101

**Published:** 2019-02-14

**Authors:** Piotr Alster, Natalia Katarzyna Madetko, Dariusz Mariusz Koziorowski, Leszek Królicki, Sławomir Budrewicz, Andrzej Friedman

**Affiliations:** ^1^Department of Neurology, Medical University of Warsaw, Warsaw, Poland; ^2^Department of Neurology, Wroclaw Medical University, Wrocław, Poland; ^3^Department of Nuclear Medicine, Medical University of Warsaw, Warsaw, Poland

**Keywords:** progressive supranucelar palsy, corticobasal syndrome (CBS), PET - positron emission tomography, SPECT and PET imaging, neuroimaging

## Abstract

Neuroimaging in the context of examining atypical parkinsonian tauopathies is an evolving matter. Positron Emission Tomography (PET) and Single Photon Emission Computed Tomography (SPECT) bring tools, which may be reasonable in supplementary examination, however cannot be interpreted as a gold standard for correct diagnosis. The review presents advantages and limitations of tau radiotracers in PET, metabolic PET and perfusion SPECT. The aim of this paper is to highlight the possibilities and boundaries in the supplementary examination of tauopathic parkinsonian syndromes.

## Introduction

Progressive supranuclear palsy (PSP) and Corticobasal Degeneration (CBD) are four repeats tauopathies which were discovered over 50 years ago. The assessment of the diseases due to their similar or overlapping symptomatology is a striking issue for neurologists, partly due to lack of adequate neuroimaging results. Current criteria of PSP released in 2017, and CBD published in 2013 demand complicated assessments (1, 2). Contemporary clinical manifestation of PSP is based on 4 basic functional domains and their level of certainty. They include *ocular motor dysfunction, postural instability, akinesia, and cognitive dysfunction* (1). Pathophysiological processes of PSP are associated with intracerebral aggregation of the microtubule-associated protein tau. In the case of PSP, the presence of the isoforms, especially in astrocytic tufts, is interpreted as the most specific (1). As PSP pathology and PSP clinical manifestation often correspond to each other, the association between CBD pathology and Corticobasal Syndrome (CBS) is not that obvious. On one hand CBS is one of four possible clinical manifestations of CBD next to frontal behavioral-spatial syndrome (FBS), non-fluent/agrammatic variant of primary progressive aphasia (naPPA) and progressive supranuclear palsy syndrome (PSPS) (2). On the other hand CBS may be a manifestation of either 3 repeat tauopathies such as Alzheimer's Disease (AD) pathology or Frontotemporal dementia pathology and 4 repeat tauopathies such as CBD and PSP. Probable and possible CBS can be related to limb rigidity or akinesia, limb dystonia, limb myoclonus, orobuccal or limb apraxia, corticobasal sensory deficit and alien limb phenomena (2). On the other hand clinical manifestation of the same CBD pathology is related to similar symptoms of typical PSP (2) These aspects lead to a search of alternative methods of examination, like positron emission tomography (PET) and single emission computed tomography (SPECT).

Although SPECT examination after application of hexamethylpropyleneamine oxime (^99m^Tc-HMPAO) and PET with 18-Fluorodeoxyglucose (^18^F-FDG) are still not interpreted as primary criteria of PSP and CBS, the evolution of those methods may result in facilitating examination (1–16). Those types of neuroimaging show regions of the brain affected by hypoperfusion or hypometabolism. Methods are not specific, additionally do not differentiate parkinsonism pathologies associated with tauopathies - PSP, CBD and synucleinopathies - Dementia with Lewy Bodies (DLB), Parkinson's Disease (PD) and Multiple System Atrophy (MSA-P) (3–6, 8–12, 14, 16, 17). Nevertheless, methods may be useful in supplementary examination and may bring factors in favor of certain diagnoses.

^18^F-AV-1451-PET is a radiotracer enabling not only differentiating tau related diseases from non-tau degenerations, but also presents individual dissemination of pathological protein within the brain (1, 18–26). Analogically, Pittsburgh-B PET can be used in the analysis of beta-amyloid deposits (21). The aim of this review is to present the examination of PSP and CBS and to show the role of SPECT and PET in the neuroimaging of those diseases. Authors of the study concentrated on the most available methods, such as perfusion SPECT and metabolic PET. The review is extended in the matter of tau radiotracers particularly due to the evolution of knowledge concerning this examination. Other radiotracers, due to their limited significance in the field, were excluded from further discussion. The authors conducted a search of available literature sources considering the matter of PET and SPECT usefulness in neuroimaging of PSP and CBS. Studies were selected on the basis of research topics considering PET, SPECT, PSP, CBS, PSP-CBS, CBD-PSP found in PubMed database. The authors searched with the following as keywords: PSP, CBS, PET, SPECT. The assumption of the literature search was to include not <65% of articles published in last 5 years, that is 2013-2018. Research studies were classified according to their relevancy.

## Tau Radiotracers in PSP

New tau-selective radiotracers include ^11^C- PBB3-PET, ^18^F-AV-1451-PET and ^18^F-THK-5351-PET (27). The accumulation of ^18^F-THK5351-PET in midbrain and basal ganglia correlates with clinical severity of PSP (27). Another study shows significant increase in accumulation of ^18^F-THK5351- in midbrain, bilateral globus pallidus, bilateral frontal cortex, medulla oblongata in PSP compared to healthy population (28). Additionally, intensity of midbrain signal correlates with the disease severity. Authors of this study, however, stress that ^18^F-THK5351-PET has also additional affinity to monoaminooxidase A and B (MAO-A and MAO-B) (28). Therefore, ^18^F-THK5351-PET is interpreted as promising, but requires further analysis correlated with autopsies (29).

^11^C- PBB3-PET, a relatively new indicator analyzed in 2013 on mice models in Alzheimer's disease, in the context of PSP examination was showed in various studies during the last years (18). In a recent study concerning PSP, where 5 patients were examined using ^11^C- PBB3-PET, increased accumulation was observed in the basal ganglia (19). In a different research, where ^11^C- PBB3-PET was compared with ^18^F-AV-1451-PET, authors indicated higher significance of ^11^C- PBB3-PET in the indication of larger variety of 4-repeat strains in comparison to ^18^F-AV-1451-PET (20).

^18^F-AV-1451-PET accumulation in midbrain and basal ganglia in PSP resembles patterns observed in healthy population in adequate age. ^18^F-AV-1451-PET besides of its binding to tau, is associated with affinity to other factors, e.g., iron deposits, iron melanin, and hemorrhagic lesions (21, 22) It should be underlined, that another study stressed affinity of ^18^F-AV-1451-PET to MAO-A (23) A different work questioned the usefulness of ^18^F-AV-1451-PET in the examination of 4 repeats (4R) tauopathies, as the authors interpreted the binding properties of ^18^F-AV-1451-PET as minimal (24). It should be also stressed that another study proved that in PSP ^18^F-AV-1451-PET affinity is not associated with severity of motor dysfunction (25). Other studies showed increased binding of the radiotracer in similar regions, however PSP was compared with Parkinson's Disease (PD) and Alzheimer's DIsease (AD) (30, 31). In PSP, compared to healthy control, an increased ^18^F-AV-1451-PET accumulation was observed in globus pallidus, putamen, subthalamic nucleus, and dentate nucleus (25). Multimodal properties of ^18^F-AV-1451-PET, like affinity to neuromelanin, led to results indicating decrease of binding of ^18^F-AV-1451-PET in the midbrain, which is associated with degeneration of neuromelanin in substantia nigra in PSP (22). However, a comparison of the results of ^18^F-AV-1451-PET and post mortem findings revealed low level of correlation of tau dissemination and results of autopsy (26). It is important to note that the increase in uptake of ^18^F-AV-1451-PET in basal ganglia can be also observed in normal aging (32). Additionally, ^18^F-AV-1451-PET does not differentiate whether the radiotracer binds to straight filaments or to helical filaments, which could also be detected in PSP (33). These factors make ^18^F-AV-1451-PET even less efficient in the examination of 4R tauopathies. ^18^F-AV-1451-PET might be more efficient in the examination of other tauopathies e.g., Alzheimer's Disease (AD) (32). The reason of different binding pattern of ^18^F-AV-1451-PET is associated with lower amounts of tau aggregates in PSP (32). Also the structure of tau aggregates differs, what is caused by various isoforms of this protein. In AD paired helical filament structures of aggregates can be observed (16). The difference in ^18^F-AV-1451-PET binding pattern between R4 and R3 tauopathies might be caused by non-homogeneous tau molecular structure in those pathologies. However, the basis of different affinity of ^18^F-AV-1451-PET between tauopathies is not fully studied.

New criteria of PSP diagnosis, define ^18^F-AV-1451-PET as a possible level 2 biomarker of PSP-RS (1, 34). Another radiotracer ^18^F-FDDNP was described as a tool in the assessment of dynamic regional localization of tau fibrillar aggregates (35).

## Tau Radiotracers in CBS

^18^F-AV-1451-PET was also assessed in CBS. In one of the studies, authors revealed asymmetrical accumulation of this radiotracer in motor subcortical gray and white matter structures, which were associated with pathology distribution (36). Another study acknowledged increased binding of ^18^F-AV-1451-PET within motor cortex, corticospinal tract and basal ganglia in CBS (37). Increased binding was observed contralaterally to the affected body side (37). It should be also noted, that authors, like in various papers concerning PSP, interpreted ^18^F- AV-1451-PET as a tracer with relatively low specificity (37). A multimodal case revealed however that ^18^F-AV-1451-PET imaging correlates with 4R-tau pathology (38). An interesting observation was made in the context of ^11^C- PBB3-PET in the examination of CBS (39). Examination using ^11^C- PBB3-PET revealed asymmetric affinity of the radiotracer, greater on the right, in the frontal, temporal, parietal, and occipital cortex (39) Analogical changes were also observed in the caudate, thalamus, globus pallidus, and ventral striatum (39).

## Non-tau Radiotracers Used in Tauopathies

Among other radiotracers, examined in the context of parkinsonian tauopathies, the assessment of dopaminergic system may have an importance. Therefore, the determination of dopamine transporter (DAT) with ^18^F-FP-CIT or the assessment of dopaminergic receptors by ^18^F-DOPA may have some importance in diagnostic procedures. Reduction of presynaptic DAT availability was observed in striatum and posterior putamen contralateral to affected side and in caudate without statistic significance of side-asymmetry in patients with CBS (40). Results of ^18^F-DOPA imaging were correlated with intensity of deficits in social behavior and behavioral manifestations in clinical examination of PSP (41). Another radiotracer ^11^C-PK11195-PET is interpreted as a diagnostic factor indicating *in vivo* microglial activation which corresponds to neuroinflammation pattern in those diseases (23, 42).

## Hypometabolism Radiotracer in PET

^18^F-FDG-PET provides evaluation of metabolism and has been found profitable in differentiation of parkinsonian disorders. Some functional domains have already been concatenated with ascendant hypometabolic brain regions, e.g., gait freezing with midbrain or unprovoked falls with thalamus (3, 43, 44). Distinctive metabolic anomaly described in PSP syndrome (PSPS) is decreased glucose consumption in the brainstem and midline frontal structures. However, hypometabolism of brainstem remains unspecific—it has been also reported in CBD or multiple system atrophy (MSA) (4). Hypometabolism of midbrain structures could be an early symptom of PSPS (45, 46), but it is not clearly visible in conventional PET images (45). Specific software provides the possibility of metabolic maps for each patient using Z-scores allowing a visual assessment of regional metabolism. Automated statistical analyses used for results elaboration, made ^18^F-FDG-PET more viable for clinical assessment, which led to an increase in the sensitivity and specificity of ^18^F-FDG-PET (4, 8). The statistical map can noticeably demonstrate the midbrain hypometabolism in PSP, which does not correlate with the severity of clinical condition, however, is classified as one of the most hope-bringing signs for an early diagnosis of PSP (45). In one of the studies a focal area of midbrain hypometabolism on ^18^F-FDG-PET scans in certain patients with PSPS (34) was described. The authors have named it “pimple sign” (47). However, its correlation with midbrain atrophy remains unclear. According to prior studies, the midbrain hypometabolism can be observed early in the course of the disease, it expands less than other hypometabolism areas and doesn't correlate with disease progression. Inversely, midbrain atrophy develops almost linearly in PSPS and correlates with clinical deterioration (4, 48). The severity of asymmetry in brain glucose uptake in cortical and subcortical motor areas correlates with the lateralization of symptoms in the course of PSP (49). The level and location of hypometabolism of cerebral cortex and basal ganglia reflects in clinical manifestation of parkinsonian syndromes (5). However it still remains difficult to distinguish between different types of atypical parkinsonian syndromes on the basis of glucose uptake, even if those data are analyzed with computer-aided methods (6).

Another study pointed out that hypometabolism in PSP assessed with 18F-FDG-PET correlates with tau pathology more significantly than examination with 18F-AV-1451 (48). Author of the study associated the results with possibly lower affinity for 4R straight filaments (50). The brain metabolic pattern in PSP patients can be characterized as highly repetitive among different populations despite of inhomogeneous methods of data analysis (multi- or univariate approach) (51). Patients with CBD present asymmetrical glucose hypometabolism, described in frontal, parietal, temporal, insular lobe and putamen, what is coherent with the neurological symptoms (8). Those findings are in agreement with preceding PET studies, which showed in patients with CBD an asymmetry in the brain metabolism with lower level of glucose consumption in the cortical and subcortical regions (7). A study conducted by Mille et al. confirmed, that patients with CBS present a significant decline of glucose uptake in the cerebral cortex (*P* < 0,01), especially in precentral and postcentral gyrus, paracentral lobule, parts of the middle frontal gyrus and the cingulate gyrus (40). Hypometabolism was also observed in subcortical regions, that is in the putamen, caudate and posterior thalamus—the asymmetry in glucose consumption (lower contralateral to the symptoms) was statistically significant (40). Niethammer et al. defined a metabolic pattern typical for CBD and named it CBDRP (corticobasal degeneration related pattern) (52). This pattern is described as asymmetrical hypometabolism (more visible contralaterally to affected body side) of the cerebrum, lateral parietal and frontal regions, thalamus, caudate nucleus with relative bilateral increases of glucose uptake in occipital regions (6). CBDRP can be helpful in diagnosing as it was specific for CBD patients when compared to healthy controls, however, it does not distinguish CBD and PSP, as in patients with PSP also CBDRP was observed (52). Possibly this metabolic pattern similarity (~24%) is caused by the overlap of brain regions covered by neurodegenerative process in this two syndromes (52). Niethammer provided an algorithm based on hemispheric asymmetry scores and expression values for PSP-related pattern, what resulted in 92,7% specificity for CBD and 94.1% specificity for PSP (52). Different patterns of glucose metabolism in parkinsonian syndromes were also described by Zhao et al. and Teune et al. (8, 9). Those results prove ^18^F-FDG-PET to be very promising method of 4-repeats tauopathies differentiation.

CBS can be a result of AD pathology. Available data confirms the effectiveness of the AD diagnosis based on ^18^ F- FDG-PET with 84 % accuracy (17). Study conducted by Zalewski et al. described presence of asymmetric parietal hypometabolism in CBD which was not observed in patients with PSP or globular glial tauopathy (GGT) (10). This study analyzes PET scans of patients with pathologically confirmed diagnoses, however, its main limitation might be a small number of analyzed patients—only 10 cases (1 CBD, 7 PSP, 2 GGT). Results published by Tripathi et al. highlight the advantages of brain metabolism assessment (visual and computer-supported) with the use of ^18^ F- FDG-PET in differential diagnosis of parkinsonian syndromes (11). Unanimity of visual evaluation of brain glucose uptake with clinical diagnosis was achieved in 91.7 % of patients, 97.6 % IPD, 80 % MSA, 76.6 % PSP, and 100 % CBS. Computer-supported assessment was effective in 91 % of evaluated PET scans with comparison to clinical diagnosis (90.4 % IPD, 80 % MSA, 93.3 % PSP, and 100 % CBS). High level of FDG-PET diagnostic accuracy when compared to clinical ≥2-years follow up results was reported by Brajkovic et al. with 92% consistence of all patients and 93%—idiopathic Parkinson's disease (IPD), 90%—multiple system atrophy (MSA), 91% - PSP and 100% CBS (12). The accuracy of PET data was 93% for IPD and MSA and 97% for PSP. Results obtained by Hellwig et al. were similar—the diagnostic accuracy of [^18^F]FDG-PET for discriminating DLB from APS and identifying specific APS subgroup was high (sensitivity/specificity 77%/97% for MSA, 74%/95% for PSP, and 75%/92% for CBD, respectively), significantly higher than [^123^I]IBZM-SPECT imaging (p = 0.0006) (53). Results obtained by Hellwig et al. highlight the advantages of performing FDG-PET on early stage of parkinsonian disorders as it can be used as predictor of survival (54). Cortical or subcortical hypometabolism detected in PET scan is an adverse predictor of lifespan which is at least as reliable as the 1-year follow-up clinical diagnosis.

## Hypoperfusion Radiotracer in SPECT

The usefulness of SPECT in the diagnosis of PSP or CBD was described in a paper published in 2001 (13). Authors of this study highlighted the patterns of frontal hypoperfusion in PSP and contralateral asymmetrical hypoperfusion in CBD (13). This study however was based on the examination of only 4 patients.

In 2017 a study conducted by Takaya et al. revealed, that combined assessment of dopamine transporter and perfusion-SPECT may be useful in differentiating neurodegenerative parkinsonian syndromes even with the lack of clinical data (14). In one of recent studies, in the context of PSP variant CBS, SPECT is presented as a supplementary tool correlating with clinical examination and magnetic resonance imaging (55). Other analyses concerning the differentiating impact of SPECT, showed possible advantages of this method in patients with clear clinical presentations of CBD, PSP or PD (15). In a different work highlighting types of dementia, PSP was indicated as a possible cause of hypoperfusion in the superior frontal cortex (16). The results were based on 5 patients with clinical diagnosis of PSP, who were examined using SPECT HMPAO (16). Another study based on examination of 2 patients using SPECT HMPAO revealed hypoperfusion in the frontal lobe (56). In a different study assessing SPECT in the differentiation of frontotemporal dementia types (FTD) 31 patients with either PSP or CBD were examined (57). The study showed that SPECT with mapping of Brodmann areas may be useful in differentiating variants of FTD (57). Another study highlighted the role of SPECT combined with neuropsychological examination in differentiation of CBS and hallucination free dementia with Lewy Bodies (58). Hypoperfusion in the occipital lobe was interpreted as a discriminating factor (58). Another analysis was conducted using a different radiotracer—[I123] lofetamine (IMP) (59). The work revealed decreased accumulation of the radiotracer in basal ganglia, superior and inferior gyrus of frontal lobe and anterior part of parietal lobe (59). Authors of the study based their conclusions on the results of 11 patients (59). A different SPECT study with iodoamphetamine based on the examination of 7 patients showed hypoperfusion in the frontal lobe (60). Two of the examined presented hypoperfusion in basal ganglia (60). A different work using the same radiotracer revealed abnormalities of perfusion in the prefrontal cortex in PSP whereas inferior prefrontal, sensorimotor, and posterior parietal cortices in CBD (61). Another study based on the differentiative abilities of SPECT in the context of CBD and PSP highlighted more significant asymmetry of perfusion in CBD (62).

## Conclusion

**Figure 1:**
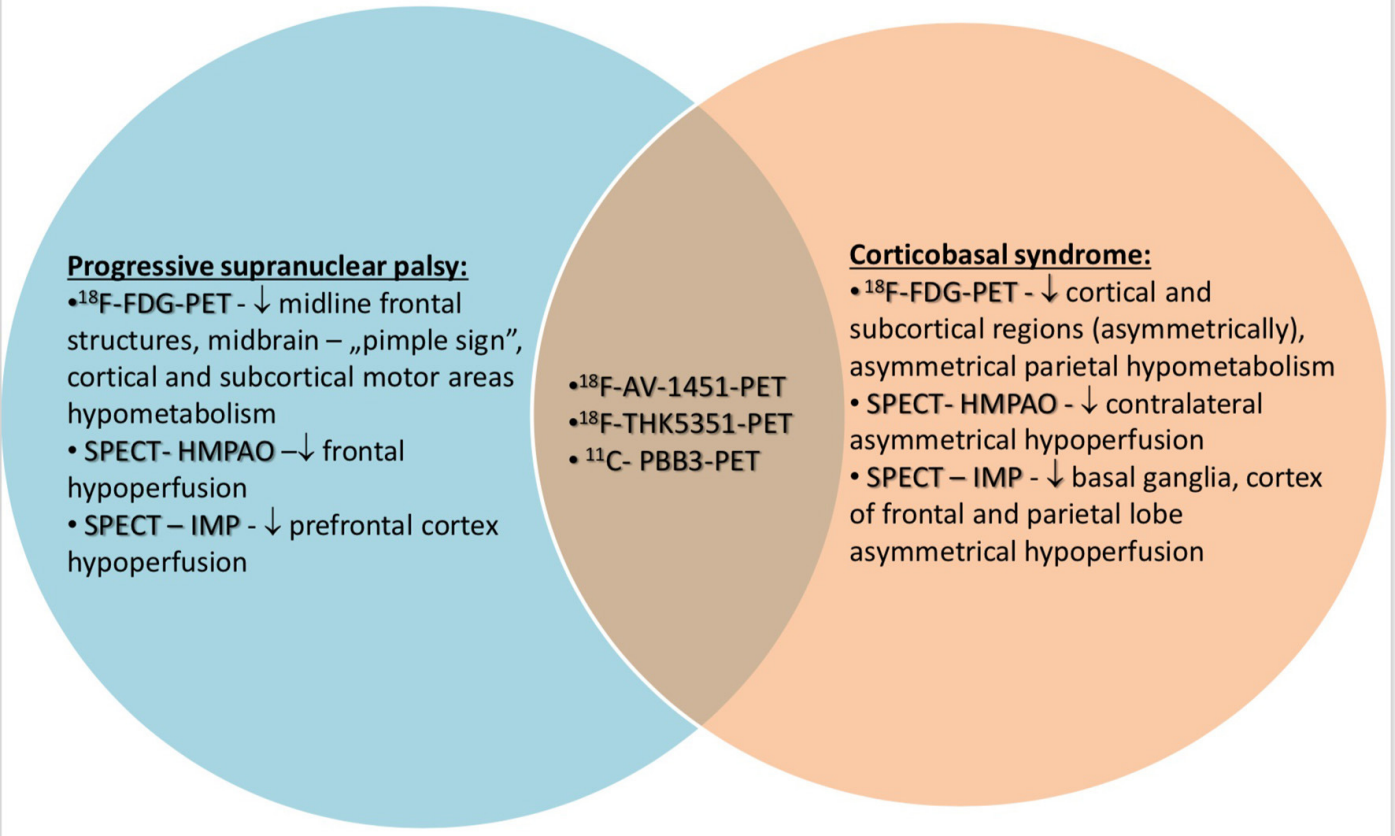
Possibilites and limitations of PET and SPECT in the examination of PSP and CBS.

Contemporary medicine is still lacking of not only optimal examination of PSP and CBS. Advances in neuroimaging based on SPECT and PET show that available radiotracers lack specificity. Tau radiotracers have relatively high affinity not only to tau, but also to monoaminoxidase A and B and neuromelanin. SPECT and the analysis of hypoperfusion is a tool which can be interpreted as supplementary to clinical examination (Figure 1). The summary of usefulness, advantages and disadvantages of radiotracers in PET and SPECT is presented in the table (Tables 1, 2). Development of neuroimaging may result in opportunities of earlier introduction of possibly effective treatment such as anti-tau antibodies or microtubule stabilizers. Further analyses of neuroimaging require longer observations and advances in specificity of accessible tools.

**Table 1:** Radiotracers–general information.

	**^**18**^FDG-PET**	**^**18**^F-THK5351-PET**	**^**18**^F-AV1451-PET**	**^**11**^C- PBB3-PET**	**SPECT-** **HMPAO/** **SPECT-IMP**
Indications	General assessment of hypometabolism, possible use in tauopathies and non-tauopathic diseases	Detection of tau in tauopathies	Detection of tau in tauopathies	Detection of tau in tauopathies	General assessment of hypoperfusion, possible use in tauopathies and non-tauopathic diseases
Advantages	-Patterns of frontal hypometabolism in PSP -Patterns of contralateral asymmetrical hypometabolism in CBS.	-Possible indication of tau -Assessment of astrocytosis -Correlation of midbrain signal with the disease severity -Increase in accumulation in midbrain, bilateral globus pallidus, bilateral frontal cortex, medulla oblongata in PSP	-Possible indication of tau -Possible level 2 biomarker of PSP-RS	-Higher than other tauopathic radio-tracers significance of ^11^C- PBB3-PET in the indication of larger variety of 4-repeat strains -Increased accumulation in the basal ganglia	-Patterns of frontal hypoperfusion in PSP -Patterns of contralateral asymmetrical hypoperfusion in CBS. -Differentiation of atypical parkinsonisms
Disadvantages	-Low specificity	-Off-target binding of MAO-A and MAO-B	-Accumulation in midbrain and basal ganglia in PSP resembles patterns observed in healthy population in adequate age. -Binding abilities associated with iron deposits, iron melanin and hemorrhagic lesions -Affinity to MAO -Affinity is not associated with severity of motor dysfunction -Does not differentiate whether the radiotracer binds straight filaments or to helical filament	-Low accessibility	-Low specificity

**Table 2:** Radiotracers – highlighted basic differences.

	**SPECT HMPAO/IMP**	**Metabolic PET**	**Tau PET**
Detection of tauopathies	Not possible	Not possible	Possible
Differentiation of parkinsonism plus	Possible	Possible	Possible only in differentiation between tauopathic and non-tauopathic parkinsonism
Accessibility	High	Moderate	Low

## Author Contributions

PA and NM: study design, data collection, data interpretation, acceptance of final manuscript version, literature search. DK, LK, SB, and AF: data interpretation, acceptance of final manuscript version.

### Conflict of Interest Statement

The authors declare that the research was conducted in the absence of any commercial or financial relationships that could be construed as a potential conflict of interest.
